# Morphology and Alignment Transition of Hexabenzocoronene (HBC) Mesogen Films by Bar Coating: Effect of Coating Speed

**DOI:** 10.1021/acs.langmuir.4c01331

**Published:** 2024-08-02

**Authors:** Hao-Chun Yang, You-Sheng Du, Jey-Jau Lee, Chun-Hong Yeh, Mei-Chun Tseng, Yi-Chi Ho, Han-Wen Kuo, Hiroyuki Yoshida, Akihiko Fujii, Masanori Ozaki, Yu-Tai Tao, Tomoyuki Akutagawg, Hsiu-Hui Chen

**Affiliations:** † Department of Molecular Science and Engineering, National Taipei University of Technology, Taipei 106, Taiwan; ‡ National Synchrotron Radiation Research Center, Hsinchu 300, Taiwan; § Institute of Chemistry, Academia Sinica, Taipei 115, Taiwan; ∥ School of Engineering Building VII, Kwansei Gakuin University, Sanda 662-8501, Japan; ⊥ Department of Electrical and Electronic Systems Engineering, Osaka Institute of Technology, Omiya, Asahi-ku, Osaka 535-8585, Japan; # Division of Electrical, Electronic and Infocommunications Engineering, Graduate School of Engineering, Osaka University, Suita, Osaka 565-0871, Japan; ∇ Graduate School of Engineering, Tohoku University, Sendai 980-8577, Japan; ○ Institute of Multidisciplinary Research for Advanced Materials (IMRAM), Tohoku University, 2-1-1 Katahira, Aoba-ku, Sendai 980-8577, Japan

## Abstract

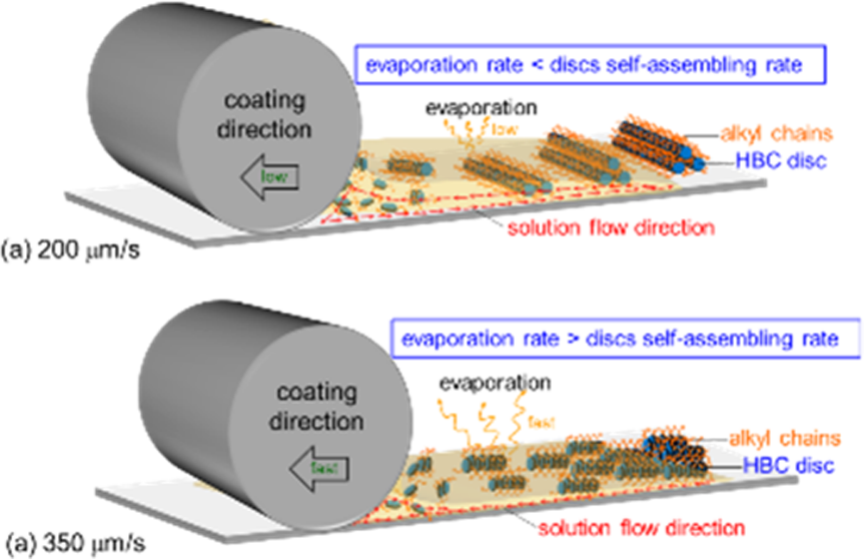

Films of the discotic liquid crystalline hexabenzocoronene (HBC) derivative, **HBC-1,3,5-Ph-C**_**12**_, were prepared on the quartz substrate by the bar-coating method. Depending on the coating speed, regularly spaced stripes or continuous films were observed. In the former case, columns of the HBC derivatives align more along the stripes, which are perpendicular to the coating direction, whereas in the latter case, columns of the HBC derivatives in the film align more along the coating direction. These distinctive structures are confirmed via polarized optical microscopy (POM), polarized UV–vis spectroscopy, and grazing incidence small-angle X-ray scattering measurements.

## Introduction

In the fields of materials science and electronics utilizing organic molecules as the active material, the controlling of molecular orientation of organic semiconductors on a substrate has been a paramount issue, since many of the material’s optical and electronic properties are direction-dependent.^1−3^ For example, for charge transport between molecules, the π–π stacking direction of a polyconjugated system is the most favored. Thus, when possible, it is desirable to have the π–π stacking direction parallel to the source/drain direction with an edge-on orientation, as in the case of field-effect transistors, or to the anode/cathode direction with a face-on orientation, as in the case of organic light-emitting diodes and photovoltaic solar cells.^4−7^ “Face-on” or “edge-on” molecular orientation depends on the interplay of intermolecular interaction, molecular-surface interaction, and surface energy consideration.^8−10^ Furthermore, in achieving in-plane direction for edge-on orientation, additional efforts are needed, such as using a prepatterned substrate on which film is formed or external force during film formation to induce the directional alignment. Various methods have been developed to induce molecular alignment, including magnetic/electric fields,^11,12^ infrared polarized lasers,^13^ solution processing,^14,15^ etc. Among these methods, solution processing is the most common and straightforward.^16−20^ Notably, the bar-coating method stands out as capable of effectively controlling the orientation of π-conjugated polymers on substrates in various directions, achieving a high conductivity. When using solution-based techniques to induce molecular orientation, factors such as solvent selection^21^ and tools employed impact the resulting molecular orientation.^22,23^ Additionally, the intrinsic aggregation ability of molecules in solution plays a pivotal role.^24−26^ In this context, discotic liquid crystalline materials (DLCs) have garnered significant attention due to their tendency to form aggregates.^27−29^

The DLCs can be categorized into high-viscosity columnar and low-viscosity nematic liquid crystalline materials. Among them, the discotic columnar liquid crystals (DCLCs), due to their high viscosity and strong π–π interactions, possess properties such as self-assembly and self-healing abilities, enabling them to automatically arrange into highly ordered rectangular or hexagonal columnar phases. They have been considered highly promising systems as charge-conducting materials if the axes of the columns can all be aligned parallel to the substrate (edge-on) or perpendicular to the substrate (face-on). Recent reports showed that by using the dewetting technique, certain DLCs can form edge-on films with their axes of columns parallel to prepatterned stripes, while with a minor variation in the structure of the discotic molecule, edge-on film with the axes of columns perpendicular to the stripes can also be obtained.^30−32^

**Figure 1:**
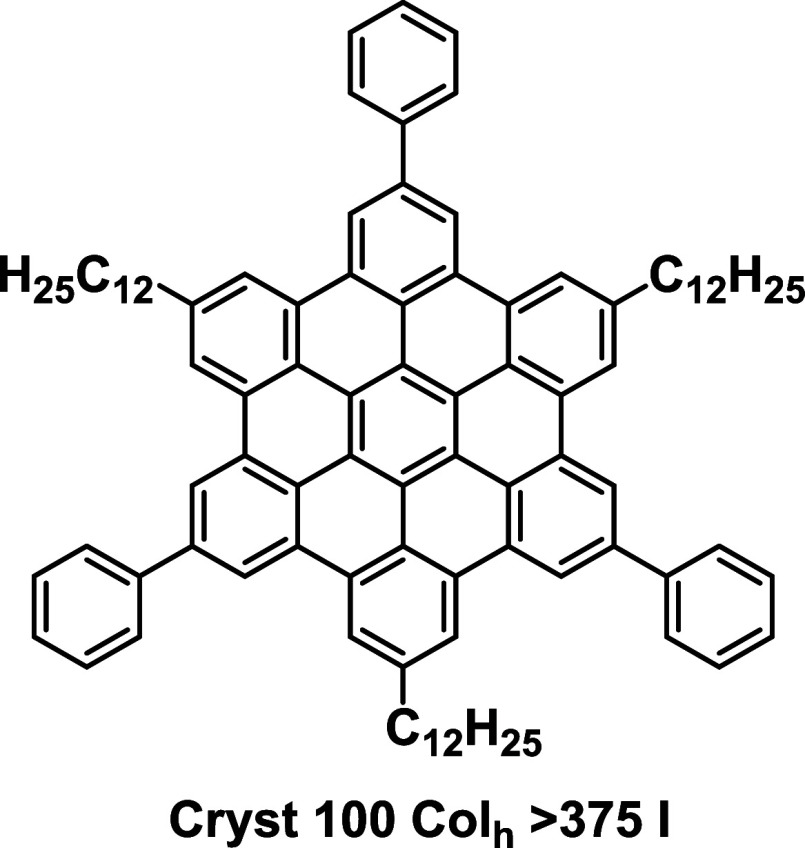
Structure of **HBC-1,3,5-Ph-C**_**12**_ used in this study. (Cryst: crystal phase, Col_h_: hexagonal liquid crystalline phase, and I: isotropic phase, unit: °C).

In this work, we chose to study the alignment of a *C*_3_-symmetry discotic liquid crystalline molecule **HBC-1,3,5-Ph-C**_**12**_ (structure shown in Figure 1)^33^ primarily because we had previously succeeded in using brush coating method to fabricate well-ordered films at the micrometer scale.^23^ However, to enable future applications in organic electronic devices, we aim to fabricate well-ordered films at the centimeter or even larger scale. Therefore, we opted for the bar-coating method in this study, which presents significantly greater challenges. It is shown that depending on the coating speed of the bar, either highly ordered and micropatterned stripes or continuous films can be obtained. For the striped film, the stripes as well as the principal axes of the columns of the HBC derivative stripes are oriented perpendicular to the coating direction. Whereas for the continuous films, the principal axes of the columns of HBCs oriented parallel to the coating direction of the bar. Detailed characterization of the structures of the stripes and films and possible causes determining the orientation and alignment are also provided.

## Experimental Section

### Materials and Methods

All chemicals and solvents were obtained commercially and used without further purification. The 2,8,14-tridodecyl-5,11,17-triphenylhexabenzo[*bc,ef,hi,kl,no,qr*]coronene (**HBC-1,3,5-Ph-C**_**12**_) was synthesized according to our previous work.^33^ It exists as polycrystalline solids at room temperature and transitions into a discotic columnar mesophase at 100 °C. Notably, the clearing point of **HBC-1,3,5-Ph-C**_**12**_ was not observed up to temperatures exceeding 375 °C. The ^1^H and ^13^C nuclear magnetic resonance spectroscopy (NMR) (Bruker, Fourier-300, Germany) spectra were recorded in CDCl_3_ and *p*-xylene*-d*^10^. Chemical shifts are in *δ* units (ppm) with the residual CHCl_3_ and *p*-xylene peaks as internal standard. The coupling constant (*J*) is reported in hertz (Hz). The optical textures and transition temperatures were observed by using a polarizing microscope (POM) (Nikon, Eclipse LV100N, Japan) equipped with a retardation plate and a Linkam T95-HS (Japan) temperature controller. Polarized UV spectra were obtained by using a UV spectrophotometer (UH5300, Hitachi, Tokyo, Japan). The orientation of the columns within the film was probed using an optical microscope with crossed polarizers (Eclipse LV100N POL, Nikon, Tokyo, Japan) and the surface morphology of the thin film was examined with an atomic force microscope (XE-100, Park, Suwon, Korea) in tapping mode. The thickness was measured by 3D laser scanning microscope (VK-X3000, Keyence, Osaka, Japan) and the water contact angle was measured by contact angle analyzer (Phoenix-300, SEO, Gyeonggido, Korea). Dynamic light scattering (DLS) spectra were taken on a particle size analyzer (90Plus PALS spectrometer, Brookhaven Instruments, New York), with the sample solution prepared in *p*-xylene and CHCl_3_. Grazing incidence X-ray diffraction (GIXRD) was measured at beamline 17 Å of the Taiwan Light Source in the National Synchrotron Radiation Research Center (NSRRC, Hsinchu, Taiwan).

### Quartz Substrate Pretreatment

The quartz plates (1.5 × 1.5 cm^2^, 99.9999%, Twicn, Taiwan) were first immersed in hexane and soapy water and sonicated for 15 min respectively to remove surface oil and impurities. Subsequently, they were rinsed thoroughly with deionized water and soaked in deionized water for another 15 min of ultrasonic cleaning. Finally, the slides were immersed in acetone and subjected to 15 min of ultrasonic cleaning. Then the slides were blown-dried with N_2_ gas and kept in drying box.

### Characterized Methods of Thin Film

**Figure 2:**
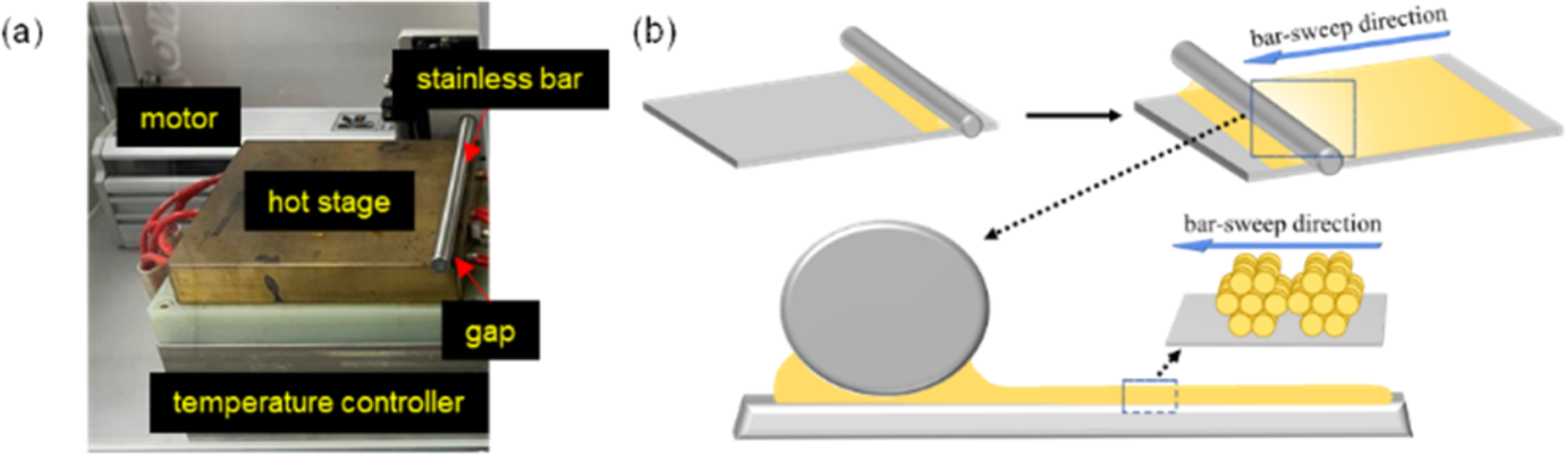
Instrument setup and bar-coating method.

The solution of HBC derivative was prepared at 1.6 × 10^–3^, 3.2 × 10^–3^, and 6.3 × 10^–3^ M in *p*-xylene and CHCl_3_, respectively. Quartz plate was placed on the stage of a custom-made bar-coater and *pre*-heated at 80, 90, 100, and 110 °C, respectively, for *p*-xylene solution, or 45 °C for CHCl_3_ solution. Then, the HBC solution in *p*-xylene was dropped on the quartz plate in front of the stainless-steel bar of the bar-coater to let the solution spread under the bar to form a meniscus on the rear side before sliding the bar at different speeds. After the thin film was formed, it was left on the hot stage for approximately 5 min to remove most of the solvent. The bar-coating process was carried out under various conditions (shown in Tables 1 and 2). The instrument setup and method for bar coating are shown in Figure 2.

**Table 1:** Effect of Different Concentrations on Dichromic Ratio

temperature (°C)	gap (μm)	speed (μm/s)	concentration (M)	dichromic ratio (*D*)
100	50	200	1.6 × 10^–3^	1.04
100	50	200	3.2 × 10^–3^	2.0
100	50	200	6.3 × 10^–3^	5.3

**Table 2:** Effect of Speed on Alignment and Directionality

temperature (°C)	gap (μm)	speed (μm/s)	concentration (M)	S_2_
100	50	50	6.3 × 10^–3^	0.24
100	50	100	6.3 × 10^–3^	0.49
100	50	200	6.3 × 10^–3^	0.69
100	50	220	6.3 × 10^–3^	0.041
100	50	230	6.3 × 10^–3^	–0.11
100	50	250	6.3 × 10^–3^	–0.23
100	50	300	6.3 × 10^–3^	–0.32
100	50	350	6.3 × 10^–3^	–0.42
100	50	400	6.3 × 10^–3^	–0.35

The orientation of the columns within the thin film was probed by using an optical microscope with crossed polarizers (Eclipse LV100N POL). The POM images of obtained **HBC-1,3,5-Ph-C**_**12**_ stripes/fibrous-films were taken in transmission mode using crossed polarizers, with the stripes oriented 0° to one of the polarizers and then rotated by 45°. The POM images were taken at ±45° to the crossed polarizers with the compensator (530 nm) in place. A retardation plate (also known as a compensator) was used to determine the alignment orientation of the DLC molecules. A first order retardation plate was placed in the optical path of the POM, with the slow axis of the retarder at +45° to the polarizer. Without a sample present, light with wavelengths on either side of 530 nm is transmitted through the analyzer, and a magenta color is seen. When a birefringent sample is observed through the microscope with the compensator in place, the spectral transmission function changes, according to the orientation of the sample. If the slow axes of the sample and the compensator are parallel, then the total birefringence is increased, and the stripe will appear blue (second order). If the slow axis of the sample is orthogonal to that of the compensator, the total birefringence is decreased and the stripe will appear yellow.^34^ The detailed setup of POM is shown in Figure S1.

## Results and Discussion

### Inherent Aggregation Ability of HBCs in Solution

First, the **HBC-1,3,5-Ph-C**_**12**_ were dissolved in *p*-xylene at 1.6 × 10^–3^, 3.2 × 10^–3^, and 6.3 × 10^–3^ M. The molecules aggregated in the solution to different extents depending on the concentration, as evidenced by the dynamic light scattering (DLS) experiments. The aggregate size distribution was determined and is shown in Figure S2. It can be seen that at a higher concentration of 6.3 × 10^–3^ M, the molecules exhibited the lowest aggregation (∼1555 nm) among the three, whereas at a lower concentration of 1.6 × 10^–3^ M, higher aggregation (∼19 882 nm) was observed. This is contrary to the general expectation and literature reports of higher aggregates at higher concentration.^35,36^ However, it was found that by replacing *p*-xylene (bp: 138.4 °C and η (viscosity): 0.34 cP at 30 °C) with nonaromatic CHCl_3_ (bp: 61.2 °C and η: 0.57 cP at 30 °C) and at 6.3 × 10^–3^ M, increased aggregation of HBC molecules with increasing concentration was observed in ^1^H NMR spectra (Figure S3). In *p*-xylene, it is evident that at high concentrations, the chemical shift moves upfield, whereas in CHCl_3_, it shifts in the opposite direction. As the degree of molecular aggregation increases, intermolecular interactions (such as π–π stacking, hydrogen bonding, etc.) alter the local magnetic environment, typically leading to a decrease in shielding effects. This causes the chemical shift to move downfield (i.e., to a higher frequency). This phenomenon is common in aromatic compounds, as aggregation changes the distribution of the electron cloud, thereby affecting the chemical shift. This suggests the crucial role played by *p*-xylene. When HBCs aggregate, the *p*-xylene may also strongly interact with HBCs, leading to more larger aggregates at low concentration. From the diffusion-ordered spectroscopy (DOSY) NMR experimental results (Figures S4 and S5), it is calculated from the peak intensity variation of aromatic rings between 6.5 and 8.0 ppm that at low concentration of 1.6 × 10^–3^ M, the diffusion rate constant is 0.32 mm^2^/s, while at high concentration of 6.3 × 10^–3^ M, the rate is 0.467 mm^2^/s. A higher value suggests smaller aggregates since they diffuse faster. These experimental results are in agreement with the DLS results presented earlier and UV–vis test in Figure S6. To further confirm whether *p*-xylene interacts with HBC molecules at different concentrations, we conducted nuclear overhauser effect spectroscopy (NOESY) and rotating frame overhause effect spectroscopy (ROESY) NMR experiments focusing on the aromatic ring of HBC at the δ 7.4 ppm position. It is evident that the signal at δ 2.07 ppm is observed at both concentrations, indicating the presence of interactions. (Figure S7) Compared to NOESY, ROESY does not experience energy decay during long scan times, making it more accurate (Figure S8). From the 1D ROESY at a low concentration of 1.6 × 10^–3^ M, the −CH_2_ signal can be seen, whereas it is absent at higher concentrations. This indirectly suggests that at low concentrations, HBC molecules packed differently from those at higher concentration. It is suggested that the alkyl chains are closer to the aromatic rings to give loose and staggered packing, which appeared to be larger aggregates. Whereas at higher concentrations, the aromatic rings stacked face-to-face (H-aggregates) and thus had closer and seemingly smaller aggregates. This indirectly proves that *p*-xylene plays a crucial role in aggregation. The high bp and η of *p*-xylene also influence the subsequent experimental results. The *p*-xylene solution was then coated on a flat quartz substrate by the bar-coating method. The gap between the bar and the substrate was set at 50 μm. A bar moving speed of 200 μm/s and a substrate temperature of 100 °C were chosen first.

### Molecular Alignment

**Figure 3:**
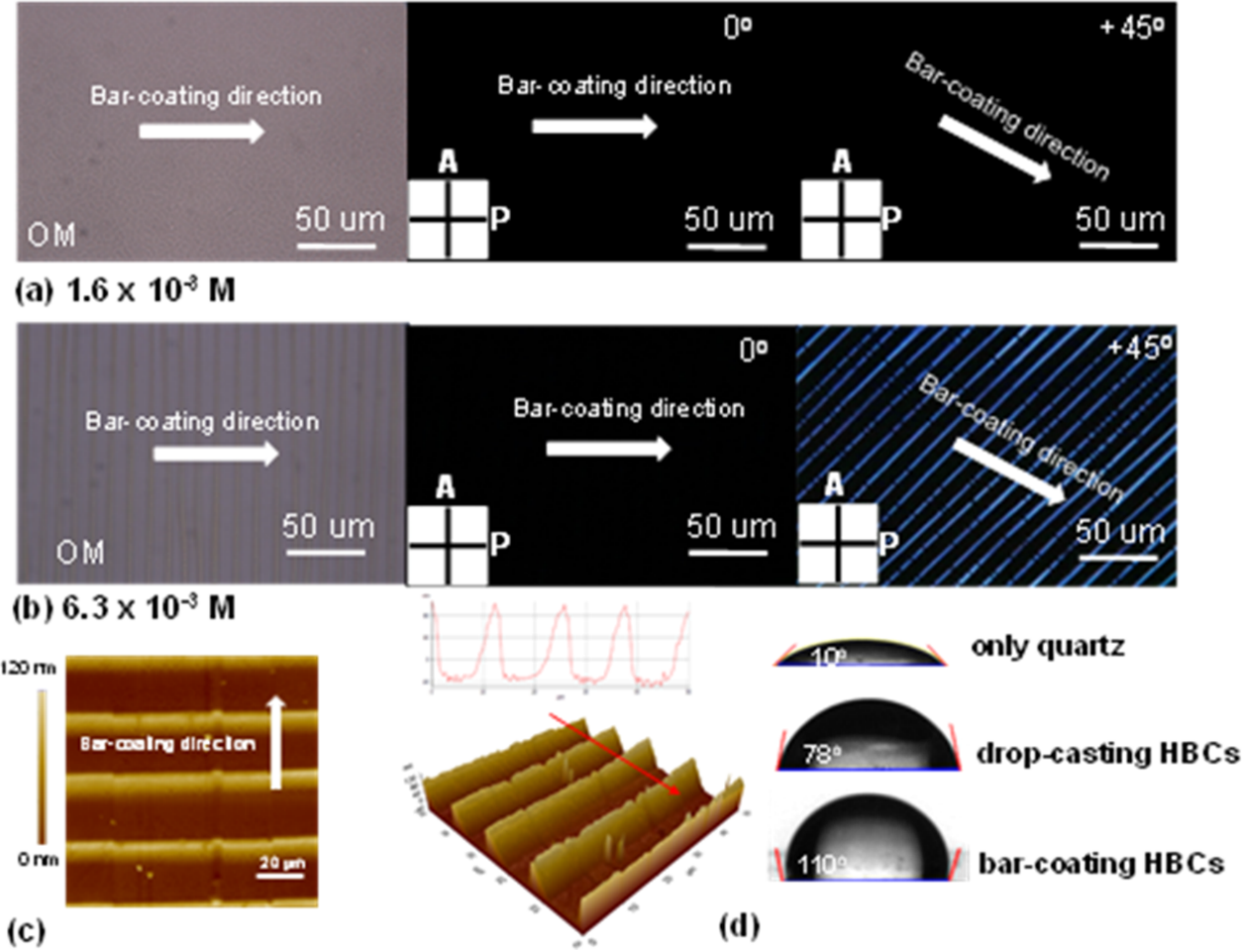
OM and POM images at (a) 1.6 × 10^–3^ M and (b) 6.3 × 10^–3^ M solution (A: analyzer, P: polarizer (A⊥P), and the top right corner indicates the rotation angle of the stage; gap at 50 μm, moving speed at 200 μm/s, hot stage *T* at 100 °C). (c) The AFM microgram of HBC film prepared as shown in panel (b). (d) Water contact angle results.

Preliminary characterization was done by OM, POM, and AFM. Based on the OM and POM results (Figure 3a,b), good molecular alignment was achieved for the solution at 6.3 × 10^–3^ M, as bright images appeared when the substrate was turned +45° from the polarizer, which suggests that the molecules tend to align well at lower aggregation states. It was also found that well-ordered and evenly spaced stripes perpendicular to the coating direction of the bar were formed, as shown in the AFM images (Figure 3c). Additionally, Figure 3c provides a profile view, revealing that each strip-like structure has nearly the same height. Subsequently, the water contact angle (WCA) was measured on a bar-coated HBC thin film. As shown in Figure 3d, with top to bottom representing a quartz plate only, drop-casted thin film, and the bar-coated thin film, the WCA is approximately 12° on bare quartz surface, around 78° on drop-casted HBC film, but 110° on bar-coated film. This indicates the formation of a very hydrophobic surface, such as one exposing mostly alkyl chains.

**Figure 4:**
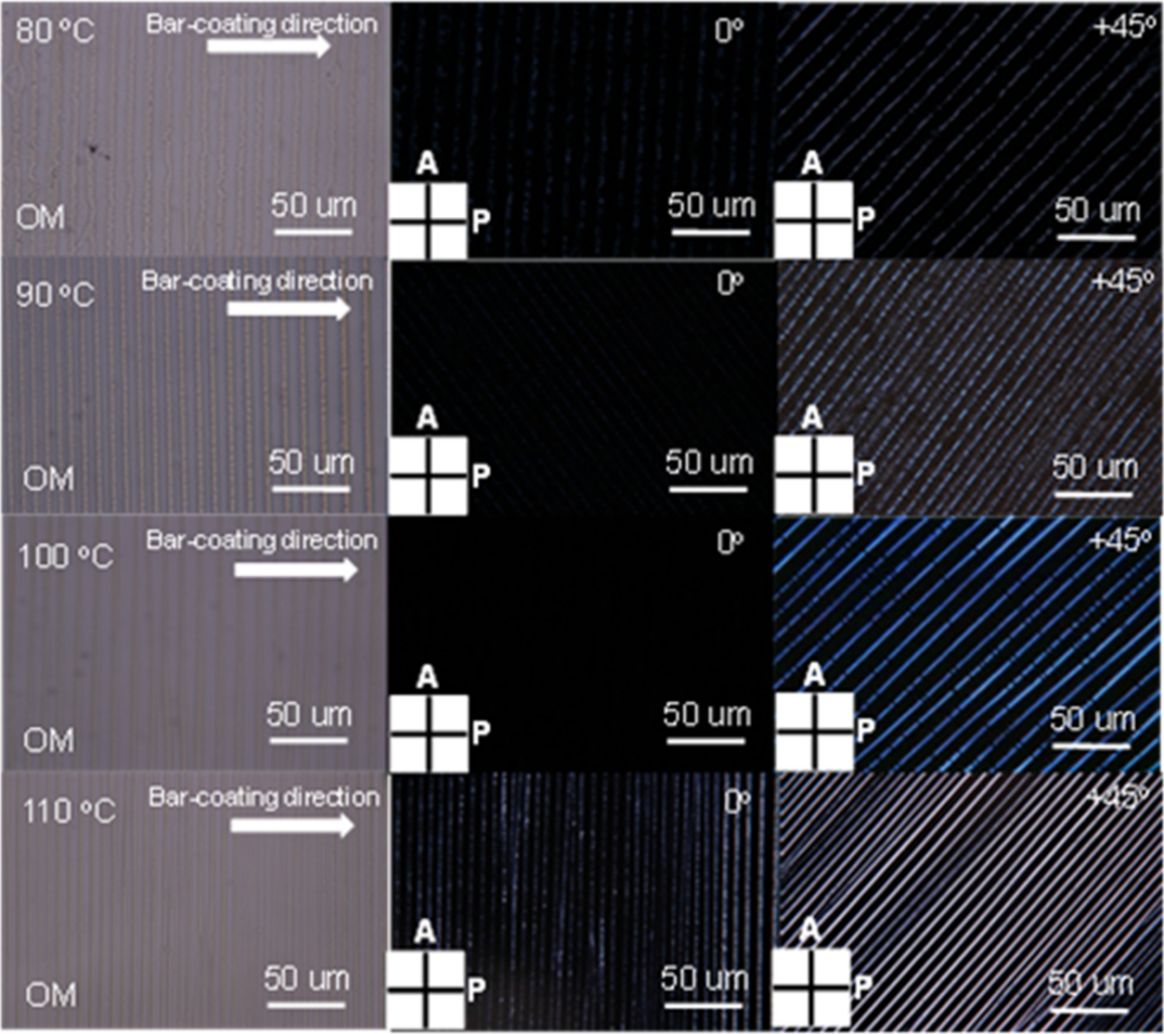
OM and POM images for films prepared at substrate temperatures of 80, 90, 100, and 110 °C. (The parameters: gap at 50 μm, moving speed at 200 μm/s, and at a concentration of 6.3 × 10^–3^ M).

**Figure 5:**
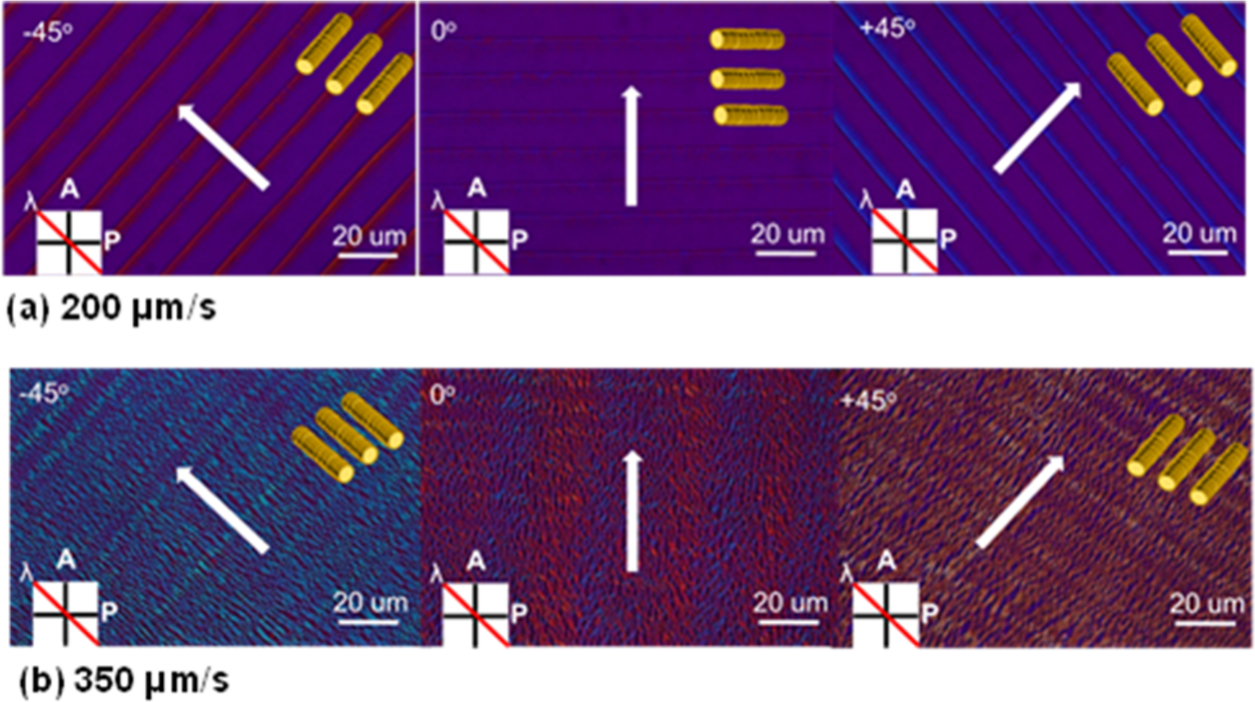
POM observations with λ plate (530 nm) to confirm the molecular alignment direction relative to the coating direction with a moving speed (a) at 200 μm/s, and (b) 350 μm/s, respectively. This was achieved by rotating the sample stage at ±45°, and 0° to the polarizer.

At 6.3 × 10^–3^ M, the effect of the substrate temperature was also examined. As shown in Figure 4, the formation of well-ordered stripes was observed for substrate temperature set at 80, 90, 100, and 110 °C, albeit with some variation in the spacing between the stripes, decreasing with increasing temperature. Thus, the solvent evaporation rate appeared to affect the spacing between the stripes. Then, the moving speed of the bar was also varied. At different moving speeds, distinct film thicknesses were observed (Table S1 and Figure S9), with a clear trend of thinner films formed at higher speeds. There also appeared to be a gradual morphology change with increasing speed. At higher speed, such as 350 μm/s, the striped structure turned into a continuous yet fibrous film, with the fibers oriented parallel to the moving direction (Figure 5). Moreover, the insertion of a λ plate with a wavelength of 530 nm between upper and lower polarizers revealed compelling support for the orientation change. Under POM, the image brightened up as the sample was turned 45° from the lower polarizer. The results show that for the striped pattern prepared at 200 μm/s, a blue color was observed with the sample stage at +45° and a red color was observed with the sample stage at −45°, indicating that the molecular columns align perpendicular to the bar-coating direction. Conversely, for the film prepared at 350 μm/s, opposite color changes were observed at ±45° of the sample stage. A transition from the striped structure to the fibrous films appeared to occur for speeds in between, where a complex morphology was observed. Most published literatures on liquid crystal polymers or highly conjugated polymers uses eq 1 to estimate their degree of alignment.^37^ In this study, we use this formula because discotic and columnar liquid crystal molecules can self-assemble into highly ordered repeating aggregates, closely resembling the alignment of polymers.^38−41^ The degree of orientation can be estimated based on the 2D order parameter *S*_2_, defined as follows
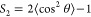
1where *θ* is the angle between the principal axial (PA) of the column direction and the bar-coating direction, and *θ* = 0 is defined to be parallel to the coating direction in this paper. When the principal axes of the column are aligned perpendicular or parallel to the bar-coating direction, *S*_2_ is positive or negative, respectively. The |*S*_2_| represents the degree of orientation, with |*S*_2_| = 0 when the principal axes of the columns are randomly oriented and |*S*_2_| = 1 when the principal axes of the columns are perfectly aligned as in a single crystal. The *S*_2_ can be calculated using an absorption dichroic ratio *D* = Abs_||_/Abs_⊥_ as
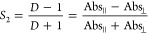
2

**Figure 6:**
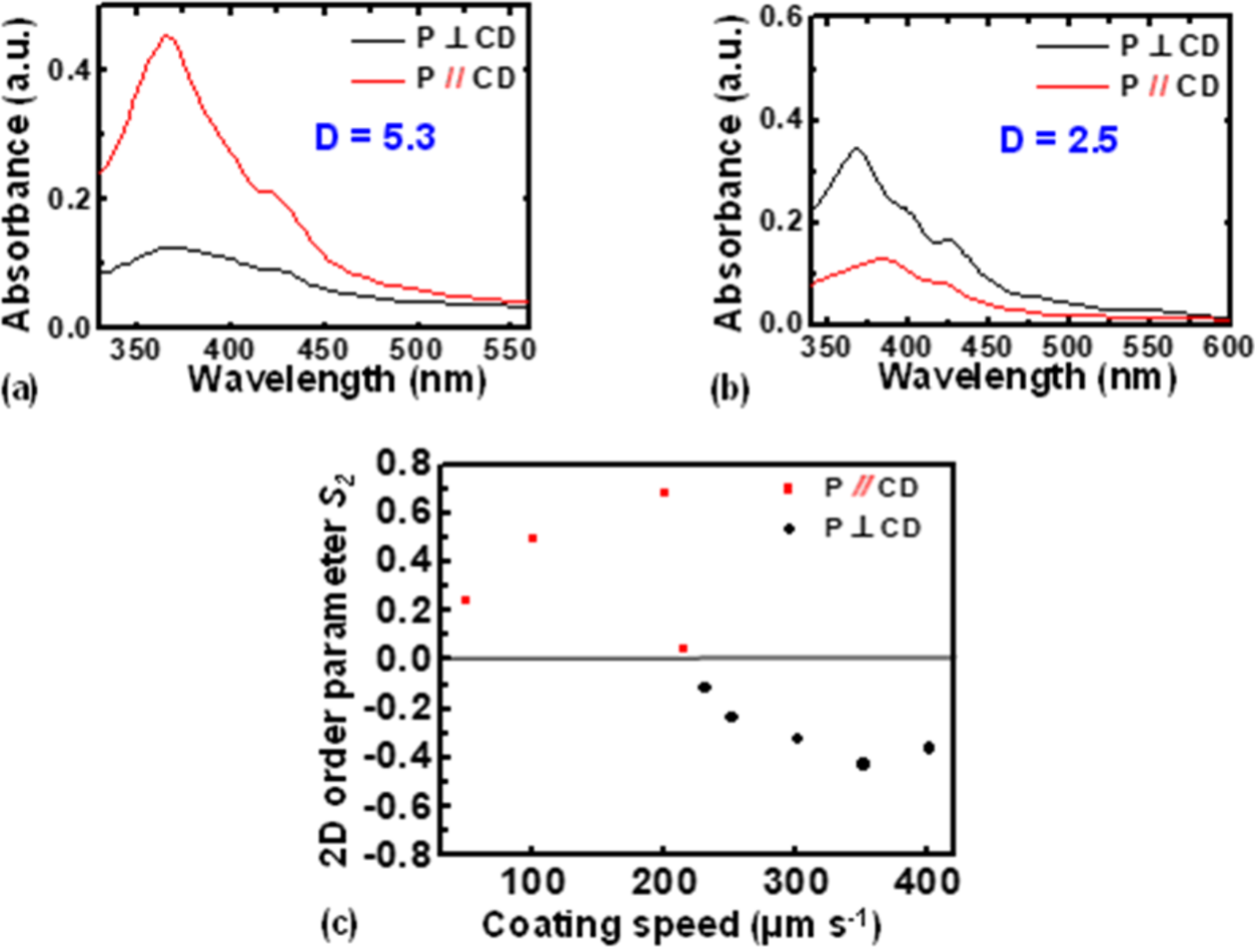
Coating speed and solution concentration dependences of 2D order parameter *S*_2_ of bar-coated thin film. The positive and negative values of *S*_2_ correspond to perpendicular and parallel orientations to the coating direction, respectively, (P is polarizer and CD is the bar-coating direction) (a) at 200 μm/s, (b) at 350 μm/s, and (c) overall at different speeds.

**Figure 7:**
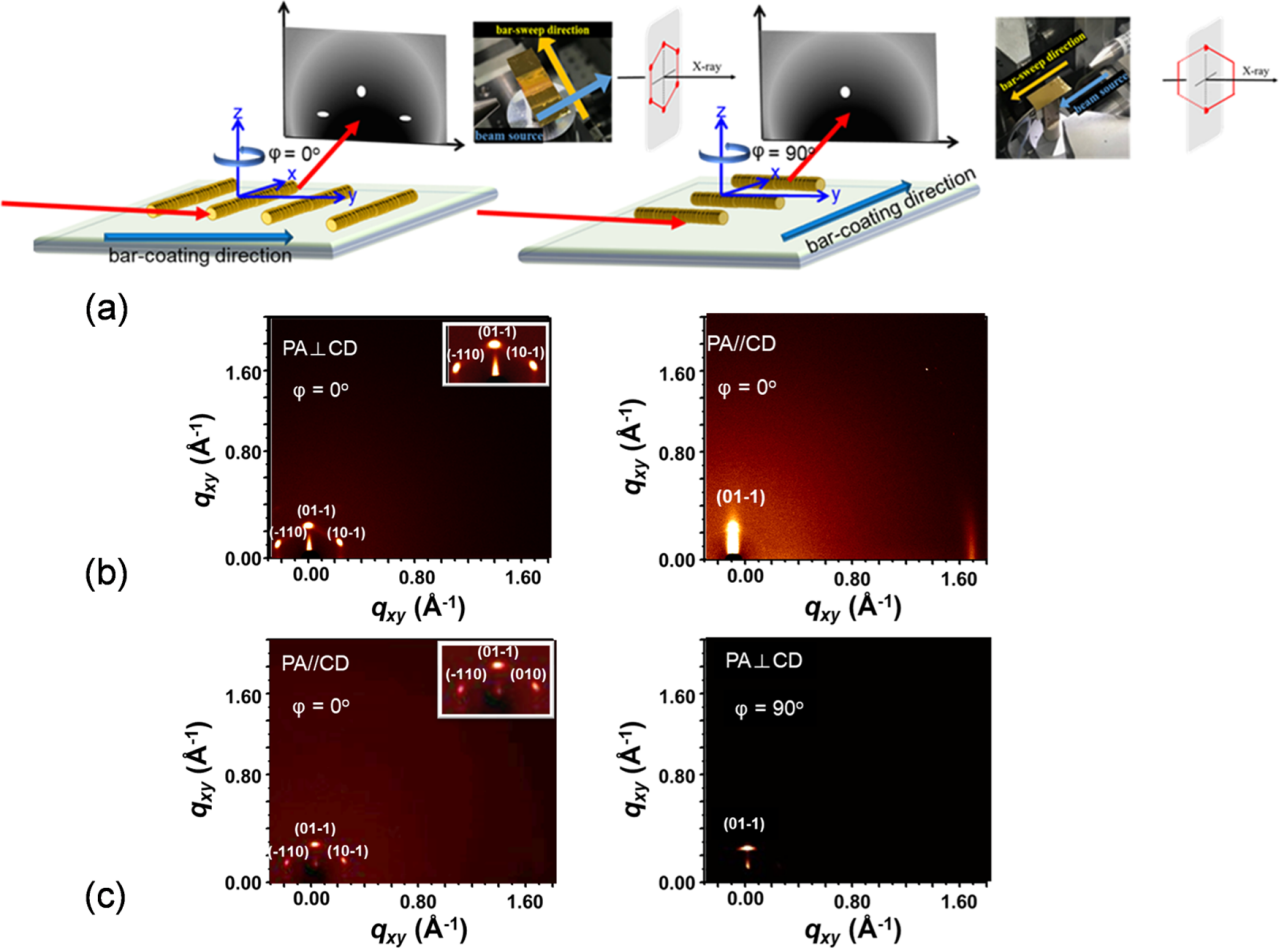
Characterizations of molecular alignment in the bar-coated HBC films. (a) The GIXRD setup. GIXRD patterns at (b) 200 μm/s with the incident X-ray beam perpendicular (φ = 90°) and parallel (φ = 0°) to the columns’ axes (c) at 350 μm/s.

As for the orientation of molecules within the films, polarized UV–vis absorption spectra were taken with the polarization perpendicular to or parallel to the coating direction. For the striped films prepared at 200 μm/s, the band peaked at 367 nm was higher when the polarization (*P*) was parallel to the bar-coating direction than that perpendicular to the bar-coating direction, with a dichroic ratio of 5.3 (Figure 6a). This indicates that the columns may have the principal axes perpendicular to the bar-coating direction. Whereas for the continuous films prepared at 350 μm/s, the trend is reversed, with a dichroic ratio of 2.5. A larger *D* value also indicates higher molecular orderliness when the molecular axis is perpendicular to the bar-coating direction, as illustrated in Figure 6b. In Figure 6c, the dichroic ratio is displayed at different speeds, the detailed experimental conditions can be seen in Figure S10. Apparently, as the speed increases from 200 to 220 μm/s, the proportion of molecular alignment along both parallel and perpendicular directions relative to the bar-coating direction becomes approximately 1:1. This indicates the coexistence of alignment in both directions, and the film thickness also decreases surprisingly from 180 to 50 nm. Therefore, this speed can be referred to as the critical speed for concentration 6.3 × 10^–3^ M in *p*-xylene. To validate the structure of the bar-coated HBC thin film, grazing incidence two-dimensional X-ray diffraction (GIXRD) measurements were conducted, and the results are shown in Figure 7. As **HBC-1,3,5-Ph-C**_**12**_ exhibits a Col_h_ phase, specific diffraction patterns can be expected depending on the column direction in the film. When the DLC columnar axis is uniformly aligned perpendicular to the incident beam, only one diffraction point (01), indicative of a layer structure, should be observed. Conversely, when the columnar axis is aligned parallel to the direction of the incident beam, the diffraction pattern should contain at least three points (10), (11), (20) providing direct evidence of hexagonal symmetry.^27^ For HBC films, two different 2D-XRD patterns (10) and (01) were observed as shown in Figure 7b,c. Although the diffraction signal of (20) was not observed, a rough hexagonal arrangement can be discerned. The possible reason for this might be the discontinuity of the columns, and the molecular arrangement model that is currently formed cannot be entirely confirmed. To indicate the differences between the left and right reflections, the Miller–Bravais formalism is used to label in Figure 7a,c. The middle reflection originates from the (01–1) plane; the left reflection is from the (−110) plane; and the right reflection is from the (10–1) plane. Modifications under different conditions may lead to the arrangement of higher-order continuous columns. It is worth noting that the highest obtained *S*_2_ value from the preceding data is only 0.62. For striped film prepared at 200 μm/s with an incident X-ray beam parallel to the coating direction, one diffraction spot was observed (φ = 90°) and with an incident beam perpendicular to the coating direction, an additional three spots were observed (φ = 0°). This would suggest the columns are perpendicular to the coating direction (or parallel to the stripes). For films prepared at 350 μm/s, just the opposite diffraction patterns were observed, indicating the columns are now parallel to the coating direction. These observations corroborate with the findings of POM and polarized UV results and confirm the alignment directions of HBCs.

Based on the above evidence, we conclude that there is a film morphology transition, from stripes with the HBC columns perpendicular to the coating direction at a lower moving speed to a continuous film with LC columns parallel to the bar-coating direction at higher coating speed. Since it is also noticed that at lower speed, the film is thicker (∼180 nm at 200 μm/s) and at higher speed, the film is thinner (∼20 nm at 350 μm/s), a possible rationale for the transition is provided here. At higher speed, the thin film crystallizes rapidly and the aggregated columns in the solution orient along the moving direction under the influence of the dragging meniscus (long axis of the column aligns with the moving solvent front). While at lower speed, the solution on the substrate is thicker and evaporating slower. Stripes are formed by a “stick-and-slip” cycles due to the surface tension effect.^42−46^ The columns in the striped solution, with all of its alkyl chains surrounding the column, tend to align along the solution edge to expose the alkyl chains to the air–liquid interface to minimize the surface energy. Upon evaporation, stripes with the LC columns orient along the solution edges, and thus, the stripes’ direction are formed. Thus, the LC columns are perpendicular to the moving direction.

## Conclusions

In conclusion, we successfully used the bar-coating method to prepare films of liquid crystal small molecule **HBC-1,3,5-Ph-C**_**12**_ onto the surface of quartz substrates, achieving an ordered columnar arrangement of the molecules. At a low speed of 250 μm/s, the molecules formed a striped morphology with the principal axes of the columnar stacks perpendicular to the moving direction of the bar. At a higher speed of 350 μm/s, a continuous and fibrous morphology was formed with the columnar stacks aligned parallel to the coating direction. These results were confirmed through polarized optical microscopy, polarized UV spectroscopy, and grazing incidence X-ray diffraction (GIXRD). The different orientations were rationalized through interplay of surface energy and flow dynamics. While similar phenomena have been reported in conjugated polymers, this is the first time such findings have been observed in organic small molecules. These discoveries provide a method for controlling the alignment of columnar liquid crystal materials and will have impact on the charge conduction in various electronic device fabrication. Further studies on their applications will be presented in our future work.

## Supporting Information Available

Instrument setup for polarized UV measurement, aggregation size distribution by DLS, aggregation behavior in different solvents and concentration by ^1^H, DOSY, NOESY, and ROESY NMR spectra, UV–vis spectra at different concentrations in *p*-xylene, POM images of films prepared at different coating speeds, dichromic ratio at different coating speeds by UV–vis spectra. (PDF)
