# Supplementary material for: Morphology and Alignment Transition of Hexabenzocoronene (HBC) Mesogen Films by Bar Coating: Effect of Coating Speed
Source: Langmuir. 2024 Aug 2;40(32):16846–54. doi: 10.1021/acs.langmuir.4c01331 (PMC11325635; doi:10.1021/acs.langmuir.4c01331)
Supplement: Supplementary file 1 — la4c01331_si_001.pdf [file la4c01331_si_001.pdf]

Electronic Supplementary Information for:

Morphology and Alignment Transition of Hexabenzocoronene (HBC) Mesogen Films by Bar Coating: Effect of Coating Speed

Hao-Chun Yang,[a] You-Sheng Du,[a] Jey-Jau Lee,[b] Chun-Hong Yeh,[c] Mei-Chun Tseng,[c] Yi-Chi, Ho,[c] Han-Wen Kuo,[a] Hiroyuki Yoshida,[d] Akihiko Fujii,[e] Masanori Ozaki,[f] Yu-Tai Tao,[c] Tomoyuki Akutagawa,[g,h] and Hsiu-Hui Chen[a]*

[a]Department of Molecular Science and Engineering, National Taipei University of Technology, Taipei, Taiwan

[b] National Synchrotron Radiation Research Center, Hsinchu City, Taiwan

[c]Institute of Chemistry, Academia Sinica, Taipei, Taiwan

[d]School of Engineering Building VII, Kwansei Gakuin University, Sanda, Japan

[e]Department of Electrical and Electronic Systems Engineering, Osaka Institute of Technology, Omiya, Asahi-ku, Osaka, Japan

[f]Division of Electrical, Electronic and Infocommunications Engineering, Graduate School of Engineering, Osaka University, Suita, Osaka, Japan

[g]Graduate School of Engineering, Tohoku University, Sendai 980-8579, Japan

[h]Institute of Multidisciplinary Research for Advanced Materials (IMRAM), Tohoku University, 2-1-1 Katahira, Aoba-ku, Sendai, Japan

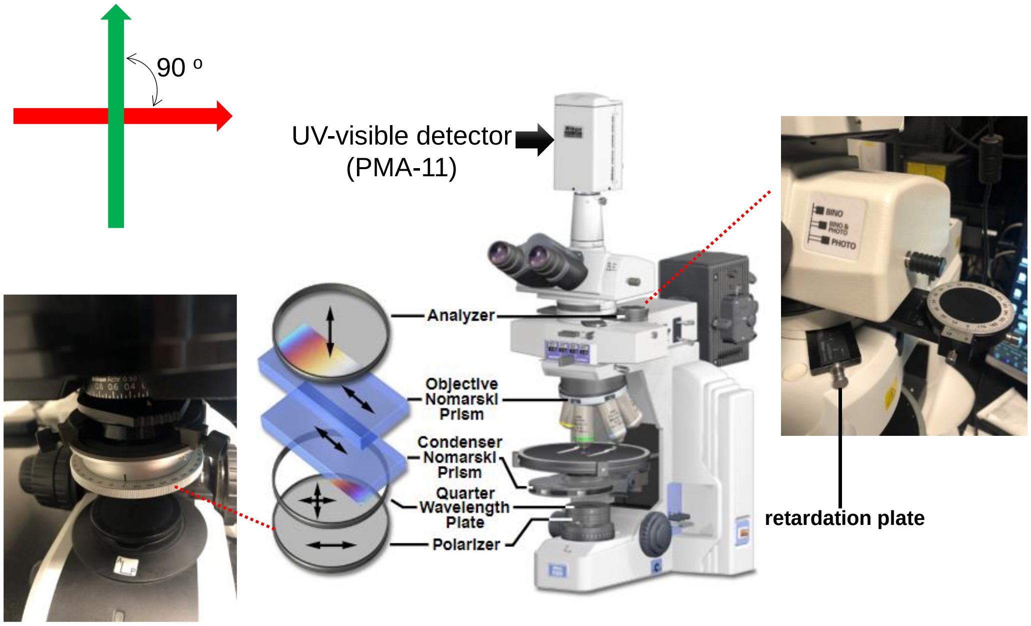

Figure S1. The instrument setup for polarized UV measurement.

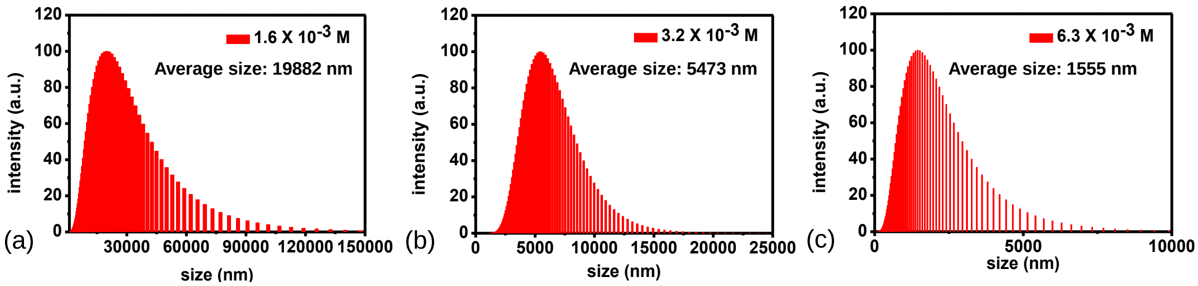

Figure S2. Aggregation size distribution of HBC derivative in *p*-xylene determined by dynamic light scattering at concentration of (a) 1.6×10^{-3} M, (b) 3.2×10^{-3} M, and (c) 6.3×10^{-3} M.

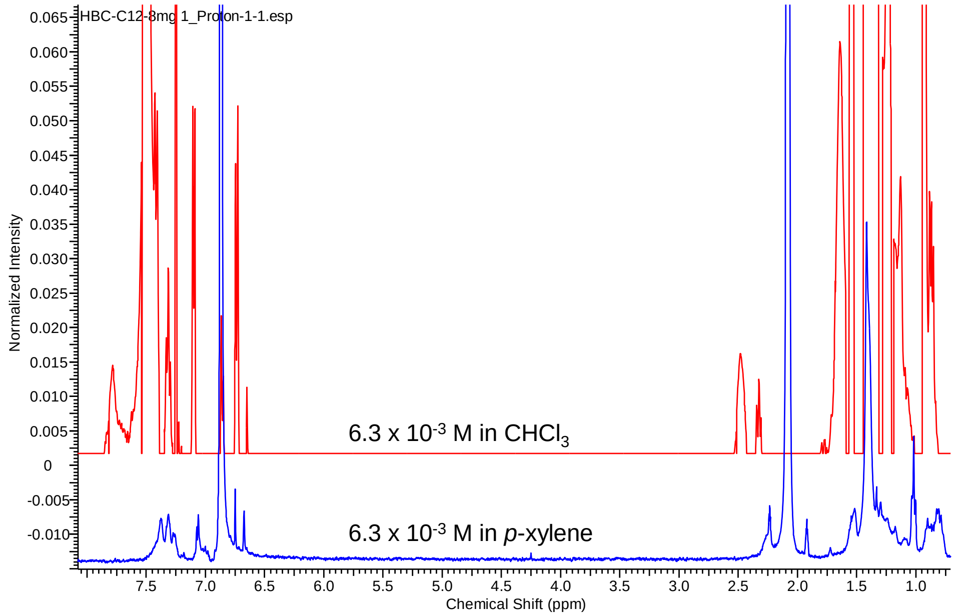

Figure S3. Using different solvents at the same concentration of 6.3 × 10^{-3} M, the observed differences in chemical shifts of **HBC-1,3,5-Ph-C_{12}** molecules are due to varying degrees of aggregation.

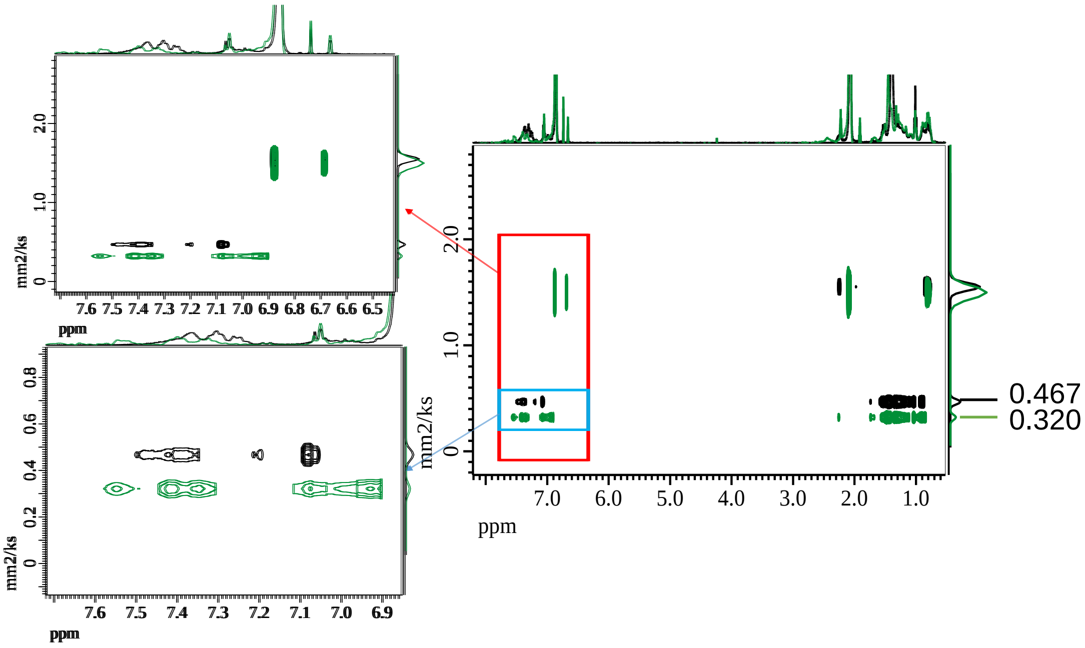

Figure S4. Overlay of the NMR DOSY spectra for **HBC-1,3,5-Ph-C_{12}** in *p*-xylene solvent at concentrations of 6.3 × 10^{-3} M (black) and 1.6 × 10^{-3} M (green).

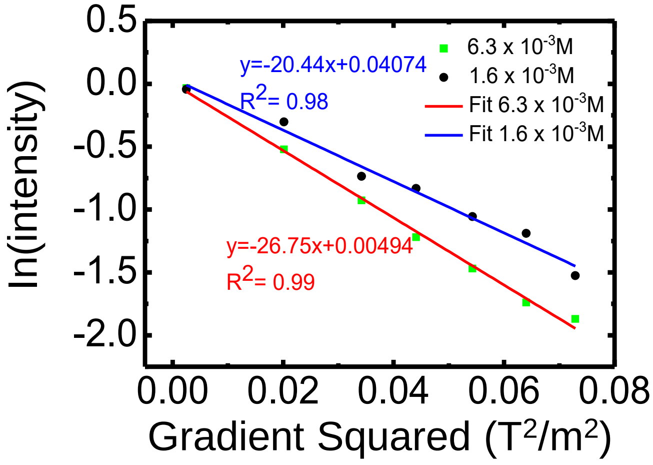

$$D=\frac{-slope}{(\gamma^2\times LD^2\times(BD-\frac{LD}{3}))}$$

γ= 2.675×10^8

LD = 0.2 ms

BD = 200 ms

Figure S5. Calculation formula the diffusion of **HBC-1,3,5-C12** in solvent is *p*-xylene.

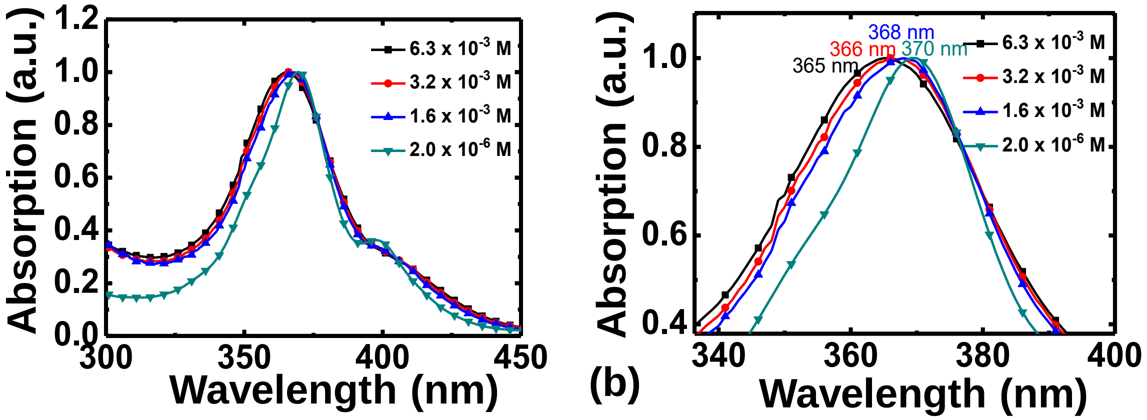

Figure S6. UV-vis spectra at different concentrations of **HBC-1,3,5-Ph-C12** in *p*-xylene.

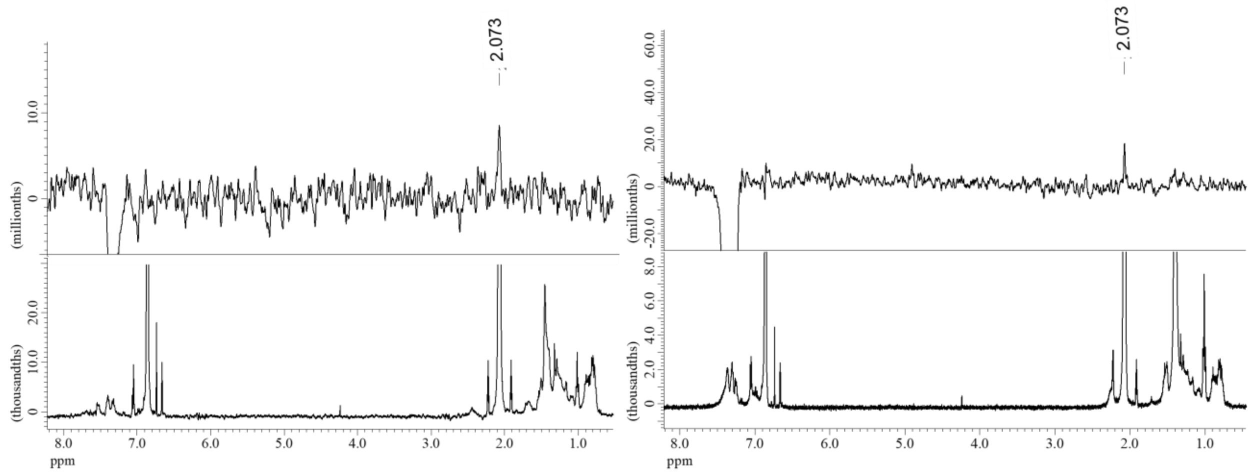

(a) 1.6 × 10^{-3} M **(b) 6.3 × 10^{-3} M**

Figure S7. NOESY and ^{1}H NMR spectra of **HBC-1,3,5-Ph-C12** in p-xylene solvent at a concentration of (a) 1.6 × 10^{-3} M; (b) 6.3× 10^{-3} M. (The upper figure shows the NOESY NMR, while the one below displays the ^{1}H NMR of **HBC-1,3,5-Ph-C12**.

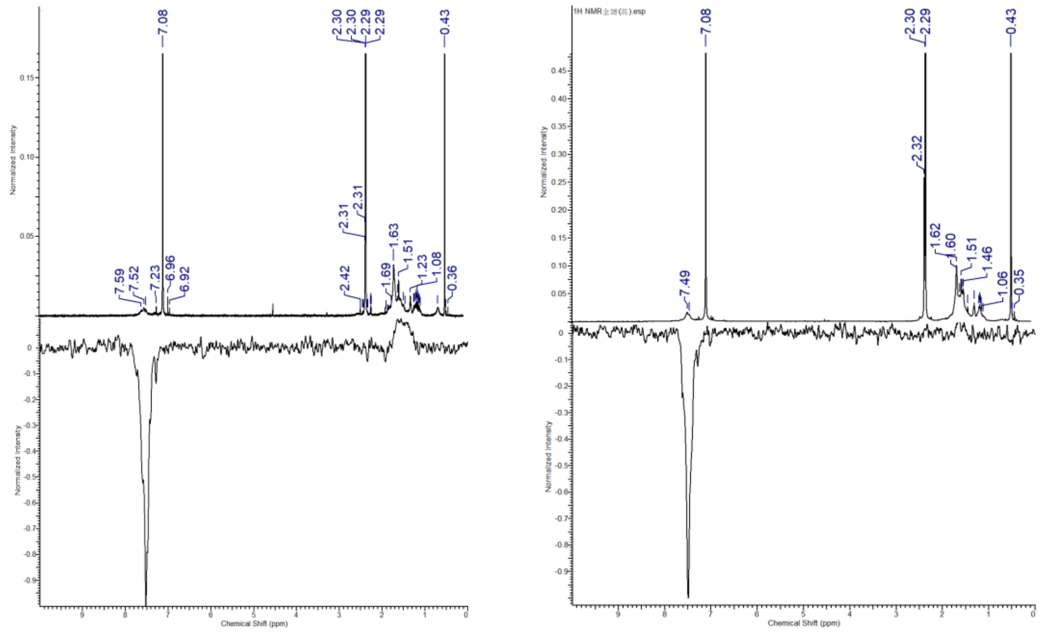

(a) 1.6 x 10^{-3} M 1D ROESY (b) 6.3 x 10^{-3} M 1D ROESY

Figure S8. ROESY NMR spectra of **HBC-1,3,5-Ph-C12** in *p*-xylene solvent at a concentration of (a) 1.6 × 10^{-3} M; (b) 6.3× 10^{-3} M.

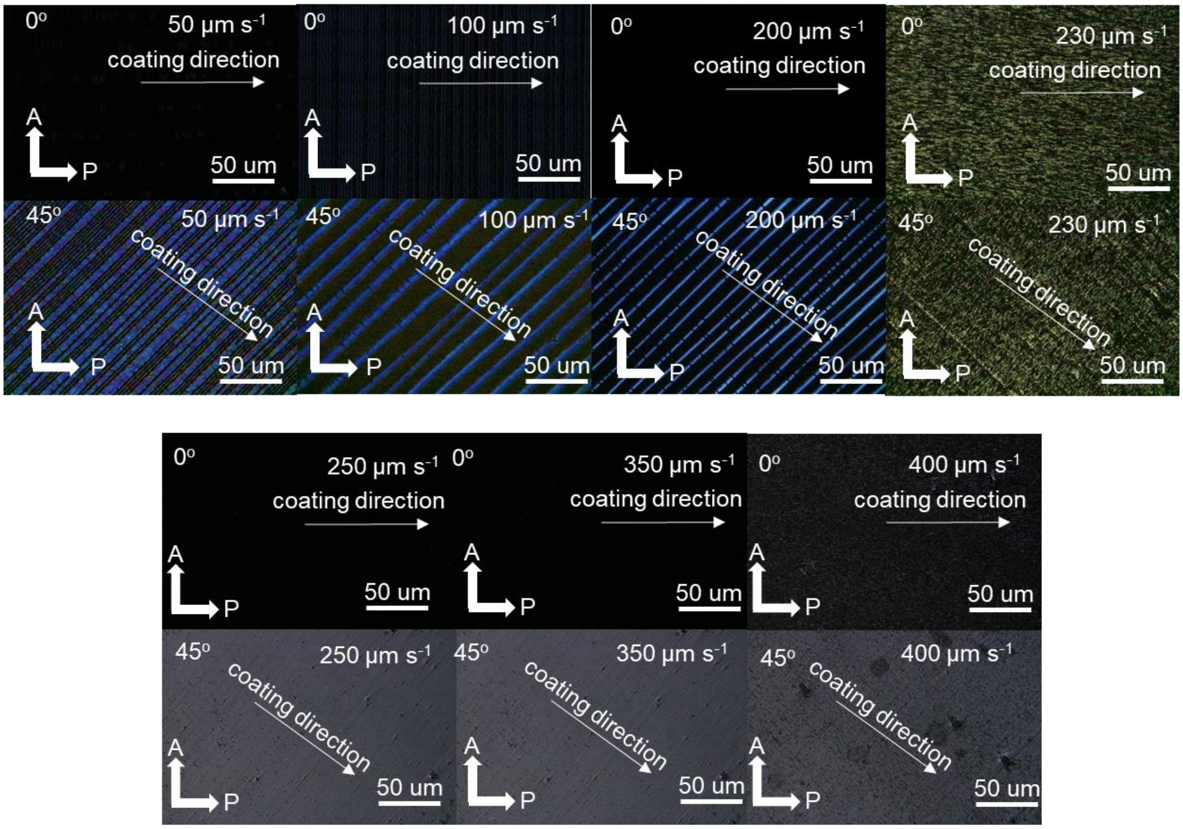

Figure S9. POM images of films prepared at different coating speeds.

Table S1. The average thickness of thin films prepared at different moving speeds.

**speeds** [µm/s]	**average thickness** (nm)
50	520
100	300
200	180
220	50
230	25
250	25
300	20
350	20
400	15

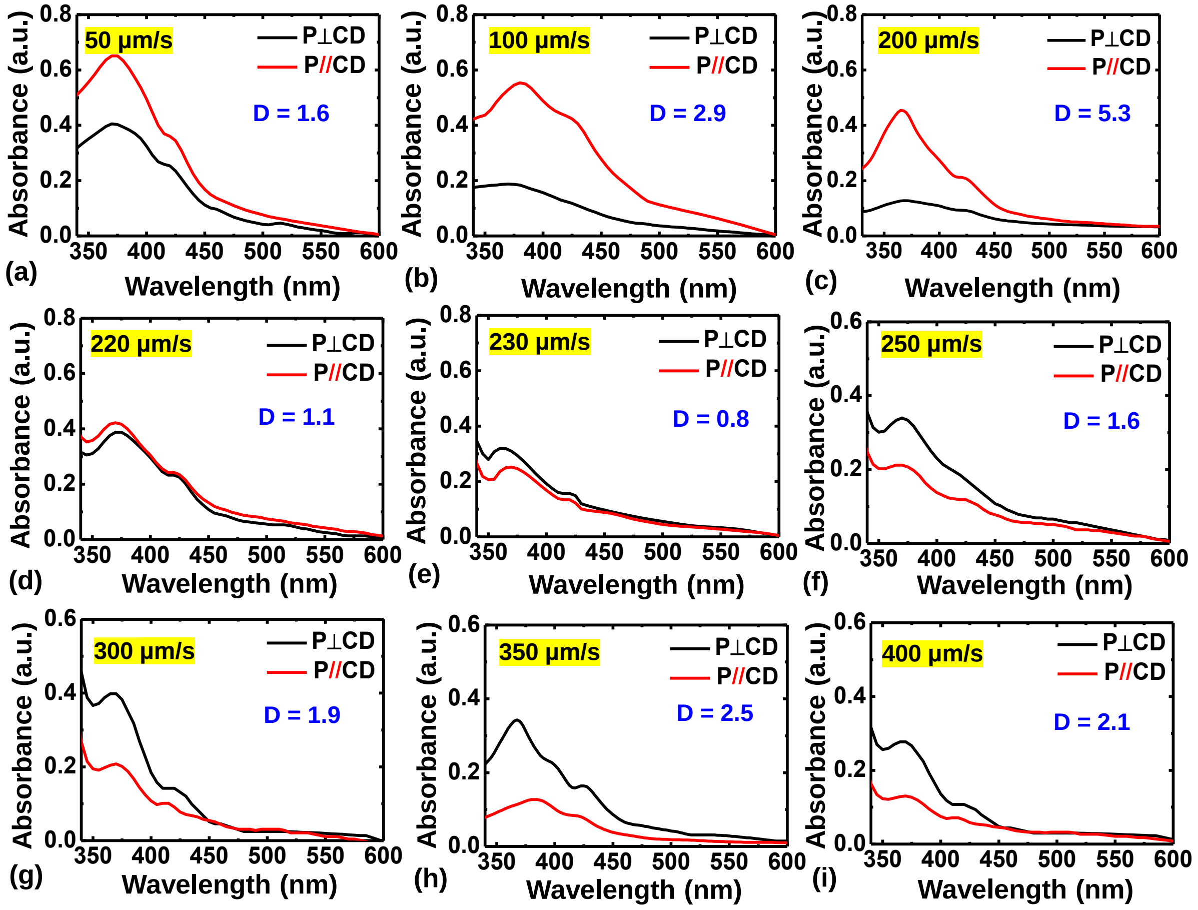

Figure S10. Dichromic ratio at different coating speeds by UV-vis spectra.
